# A Computer-Aided Heart Valve Disease Diagnosis System Based on Machine Learning

**DOI:** 10.1155/2023/7382316

**Published:** 2023-01-19

**Authors:** Si-ji Ding, Hao Ding, Meng-fei Kan, Yi Zhuang, Dong-yang Xia, Shi-meng Sheng, Xin-ru Xu

**Affiliations:** ^1^School of Health Science and Engineering, University of Shanghai for Science and Technology, Shanghai 200093, China; ^2^School of Medical Devices, Shanghai University of Medicine and Health Sciences, Shanghai 201318, China

## Abstract

Cardiac auscultation is a noninvasive, convenient, and low-cost diagnostic method for heart valvular disease, and it can diagnose the abnormality of the heart valve at an early stage. However, the accuracy of auscultation relies on the professionalism of cardiologists. Doctors in remote areas may lack the experience to diagnose correctly. Therefore, it is necessary to design a system to assist with the diagnosis. This study proposed a computer-aided heart valve disease diagnosis system, including a heart sound acquisition module, a trained model for diagnosis, and software, which can diagnose four kinds of heart valve diseases. In this study, a training dataset containing five categories of heart sounds was collected, including normal, mitral stenosis, mitral regurgitation, and aortic stenosis heart sound. A convolutional neural network GoogLeNet and weighted KNN are used to train the models separately. For the model trained by the convolutional neural network, time series heart sound signals are converted into time-frequency scalograms based on continuous wavelet transform to adapt to the architecture of GoogLeNet. For the model trained by weighted KNN, features from the time domain and time-frequency domain are extracted manually. Then feature selection based on the chi-square test is performed to get a better group of features. Moreover, we designed software that lets doctors upload heart sounds, visualize the heart sound waveform, and use the model to get the diagnosis. Model assessments using accuracy, sensitivity, specificity, and *F*1 score indicators are done on two trained models. The results showed that the model trained by modified GoogLeNet outperformed others, with an overall accuracy of 97.5%. The average accuracy, sensitivity, specificity, and *F*1 score for diagnosing four kinds of heart valve diseases are 98.75%, 96.88%, 99.22%, and 97.99%, respectively. The computer-aided diagnosis system, with a heart sound acquisition module, a diagnostic model, and software, can visualize the heart sound waveform and show the reference diagnostic results. This can assist in the diagnosis of heart valve diseases, especially in remote areas, which lack skilled doctors.

## 1. Introduction

Cardiovascular diseases (CVDs) are one of the leading causes of death each year, causing an estimated 17.9 million deaths each year, according to the statistical data of the World Health Organization (WHO) [1]. It includes coronary heart disease, valvular heart disease, rheumatic heart disease, and other conditions, and valvular heart disease accounts for about 20% of cases, which is a large contributor to the burden of disease [2]. There are four heart valves, namely the aortic, mitral, tricuspid, and pulmonary valves. Heart valves will close alternately to prevent the regurgitation of blood, and the sound of valve closure can be heard using a stethoscope. If the valve is abnormal, it will reflect in the heart sound. A cardiologist can diagnose heart valve diseases by auscultation based on murmurs of heart sounds.

Since 2020, due to the pandemic of COVID-19, medical staff should wear a protective suit to prevent infection of COVID-19 when carrying out treatment and diagnosis. It is no longer possible to use a conventional stethoscope. Some experts suggested using handheld ultrasonic devices instead of a stethoscope [3]. Although an ultrasound examination can provide more information on heart conditions, it requires ultrasonic equipment and skilled cardiologists to interpret the images. In conclusion, auscultation is of great value in the diagnosis of valvular heart disease.

However, auscultation has very high demands of professionalism for clinicians, and it takes a long time to master auscultation techniques, which can only be improved by practising on a large number of patients [4]. Moreover, it is more difficult to train a doctor expert in auscultation in developing areas than in developed areas.

Since machine learning is more and more used in medical fields [5], it is helpful to use machine learning for heart disease diagnosis based on heart sounds.

There are mainly three steps in training a model to diagnose heart valve diseases by machine learning: (i) signal preprocessing, (ii) feature extraction and selection, and (iii) classification. Generally, the signal preprocessing step includes signal denoising and segmentation. Then features will be manually extracted or used with the neural network to be automatically extracted. Finally, the model will be trained by machine learning algorithms to diagnose diseases.

In the early years, many researchers emphasized signal segmentation to divide heart sounds into S1 (the first heart sound), systolic, S2 (the second heart sound), and diastolic segments [6–11]. However, accurate segmentation relies on the ECG signal to locate the boundaries of heart sounds. Other segmentation methods based on signal processing such as Shannon energy and average power spectrum, cannot access accurate segmentation, which will have a negative effect on classification. Recently, studies have shown that extracting features directly without segmentation can also achieve good performance on classification [12–16]. Thus, we proposed a model without segmentation.

Table 1 shows some related works on heart sound classification.

In other studies, heart sounds are usually classified into normal and abnormal categories, which cannot be used to diagnose specific heart diseases [45, 46]. In this study, we have trained two models based on a convolutional neural network and a classic machine learning algorithm weighted *k*-nearest neighbour to diagnose heart valve diseases. The two models are assessed using given indicators, including accuracy, specificity, sensitivity, and *F*1 score, and the model trained by modified GoogLeNet was selected after model assessment. It can automatically classify the heart sounds of healthy people and different valvular conditions, which are aortic stenosis (AS), mitral stenosis (MS), mitral regurgitation (MR), and mitral valve prolapse (MVP).

In addition, we designed a computer-aided heart valve disease diagnosis system. The framework of this system has four parts: collecting heart sounds and uploading the audio file; preprocessing the signal; diagnosing based on the trained classification model; and showing the diagnosis on the app. We not only trained a model with high accuracy of valvular heart disease classification but also designed a diagnostic system, which can assist in clinical diagnosis.

## 2. Materials

**Figure 1:**
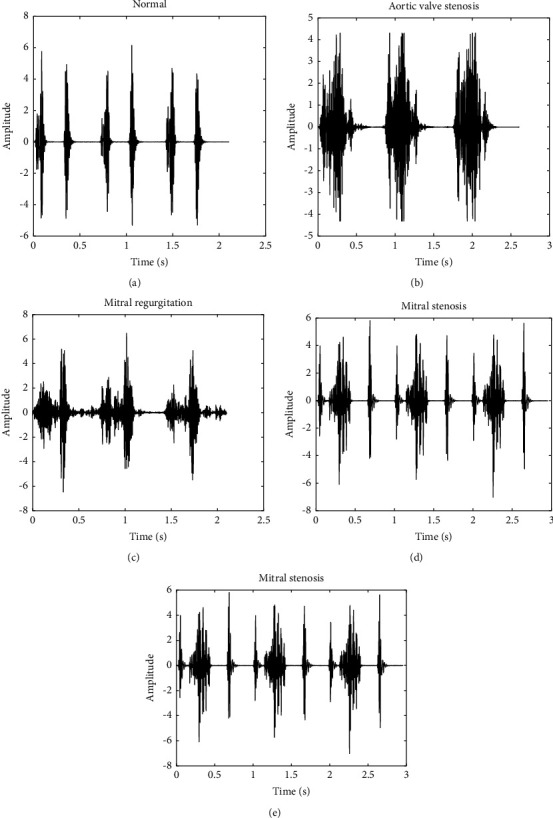
Heart sound signal. (a) Normal heart sound signal. (b) Aortic valve stenosis. (c) Mitral regurgitation. (d) Mitral valve prolapse. (e) Mitral stenosis.

The heart sound data in this study were obtained from a public dataset [21]. This heart sound dataset contains 1000 heart sound audios of 5 classes: normal heart sound, aortic stenosis (AS), mitral stenosis (MS), mitral regurgitation (MR), and mitral valve prolapse (MVP). Each of the heart sound signal is sampled at 8000 Hz.  Figure 1 shows an example of 5 classes of heart sounds.

## 3. Methods

### 3.1. Transfer Learning for Heart Valve Disease Diagnosis

Training deep neural networks usually requires a large number of data sets and computing resources. This study has only 1000 heart sound data, which is too small for a deep neural network to train the model. This may affect the accuracy of the model. In this case, we can use the pretrained network to train the model, a method called transfer learning.

GoogLeNet is a convolutional neural network for image classification, which is based on the inception architecture. It has 22 layers with 9 inception modules. Each inception module contains different sizes of convolution kernels from different sizes of 1*∗*1 to 5*∗*5 and a pooling layer. Even for the same image, the size of the convolution kernels will affect the effect of convolution. By utilizing the inception architecture, the network can choose whether to use a bigger or smaller size of convolution kernel by adjusting different weightings. So, it can classify images with high accuracy.

**Figure 2:**
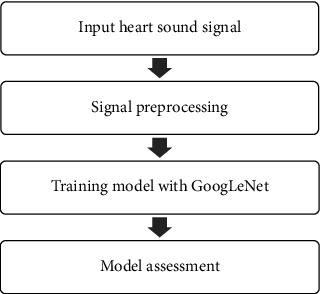
Flowchart of heart valve disease classification based on heart sound signal.

In this study, GoogLeNet was used for training the heart valve disease classification model, mainly including the following steps, as shown in Figure 2.

#### 3.1.1. Signal Preprocessing

GoogLeNet requires RGB input images with a size of 224-by-224-by-3. To meet this requirement, we have to preprocess the heart sound signal, which is a one-dimensional time series. A time-frequency representation is used to transfer a one-dimensional heart sound signal to a scalogram, which is a three-dimensional RGB image [22, 47].

Before transforming heart sound signals into images, signal normalization has been done using equation (1), where *μ* and *σ* is the mean and standard deviation of the signal. The time-frequency representation is based on the continuous wavelet transform (CWT). The steps of transforming heart sound signals to a scalogram are (i) create a CWT filter bank of the signal based on the Morse wavelet; (ii) perform CWT using the filter bank created in Step 1 to get the scalogram; and (iii) resize the scalogram as 224-by-224-by-3 to fit the input size of GoogLeNet.

(1) $$\begin{array}{c} x_{new}=\frac{x-\mu }{\sigma }\text{.} \end{array}$$

**Figure 3:**
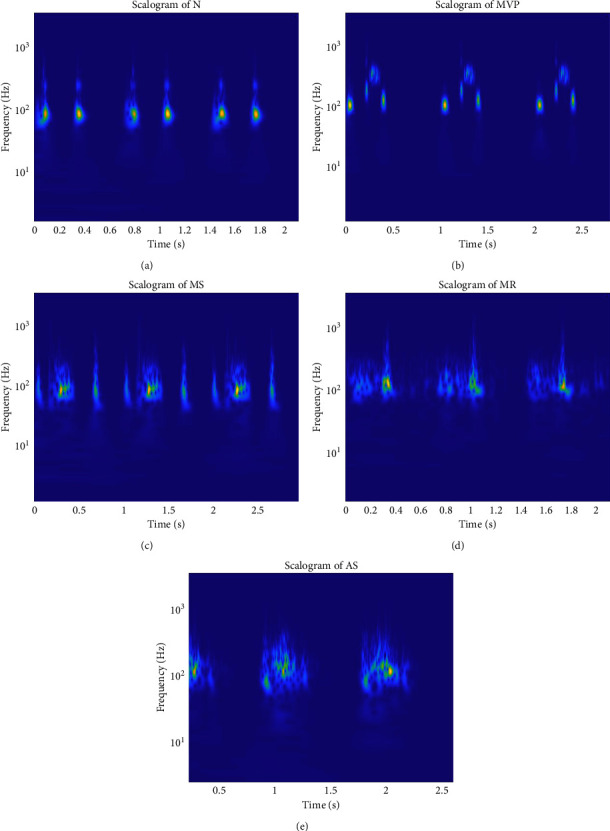
Scalograms of 5 categories of heart sound signals. (a) Scalogram of the normal heart sound. (b) Scalogram of heart sound with mitral valve prolapse. (c) Scalogram of heart sound with mitral stenosis. (d) Scalogram of heart sound with mitral regurgitation. (e) Scalogram of heart sound with aortic stenosis.

The scalogram is a three-dimensional image whose horizontal axis represents time, the vertical axis represents frequency, and the colour represents the magnitude of the corresponding frequency and time. The scalograms of 5 categories of heart sound signals are shown in Figure 3.

After collecting the scalograms of all categories of heart sound signals, they are divided into two parts, which are the training set and the validation set. The former is used to train the model, and the latter is used to verify whether the model performs well. In this case, there are 800 scalograms in the training set and 200 scalograms in the validation set.

#### 3.1.2. Training Model with GoogLeNet

GoogLeNet is a kind of CNN, which is designed for image classification. It is widely used in medical image classification tasks [48]. However, it is seldom used in heart sound classification tasks because GoogLeNet requires the input of 224-by-224-by-3 RGB images, while heart sound signals are one-dimensional time series.

In this case, we transform the original heart sound signals into scalograms to adapt the pattern of GoogLeNet. Besides, GoogLeNet is designed to classify 1000 categories of images, so we tuned some parameters of a few layers, such as the fully connected layer and output layer, to make the network match our training set. Firstly, we adjusted the dropout probability to 0.6 of the final dropout layer in the neural network. Secondly, we change the output size of the fully connected layer “loss3-classifier” to 5, which corresponds to the categories of our dataset. Thirdly, we replace the output layer with the classification layer to classify different categories of heart sounds.

**Figure 4:**
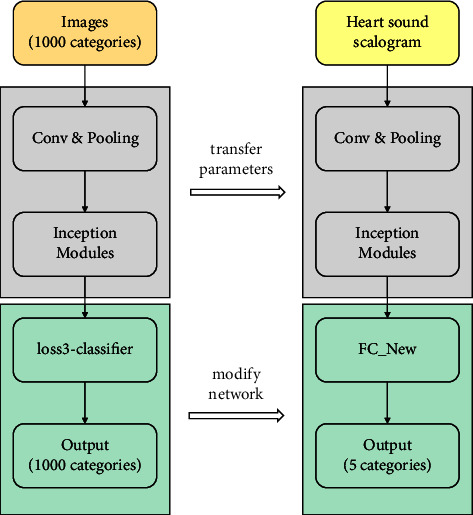
Modified GoogLeNet architecture.

Other options for the neural network are set as follows: we set the learning rate to 0.0001, mini-batch size to 15, and max epoch to 20. At last, the stochastic gradient descent with the Momentum optimizer is used for optimization. The modified architecture of GoogLeNet is shown in Figure 4.

### 3.2. Classic Machine Learning Algorithm

Classic machine learning algorithms include K nearest neighbour (KNN), support vector machine (SVM), decision tree (DT), etc. It is different from the neural network in that those classic machine learning algorithms mentioned above require manually extracting features from signals and then using algorithms to train the model. The performance of a model may be highly impacted by feature selection.

The main steps of training a heart valve disease diagnosis model using classic machine learning algorithms include feature extraction, feature selection, classification, and model assessment, which are introduced in the following sections.

#### 3.2.1. Feature Extraction

Heart sound signals are one-dimensional time series. Time and frequency domain features are extracted manually in this step. A total of 26 features are extracted, which include 10 time domain features and 16 frequency domain features. The extracted features are listed in Table 2.

In the time domain, 10 features are extracted manually. RMS is the square of the mean square of the signal. The shape factor can be calculated by dividing the RMS by the mean of the absolute value. The skewness and kurtosis are the third and fourth moments of the signal, which are shown in equations (2) and (3). The peak value is the maximum absolute value of the signal. The impulse factor is given by the peak value divided by the mean value of the absolute value of the signal. The crest factor is given by the peak value divided by the RMS. The clearance factor is given by the peak value divided by the squared mean value of the square roots of the absolute value of the signal.

(2) $$\begin{array}{c} Skewness=\frac{1/N\overset{N}{\underset{i=1}{\sum }}{\left(x_{i}-\bar{x}\right)}^{3}}{{\left(1/N\sum_{i=1}^{N}{\left(x_{i}-\bar{x}\right)}^{2}\right)}^{3/2}}\text{,} \end{array}$$

(3) $$\begin{array}{c} Kurtosis=\frac{1/N\overset{N}{\underset{i=1}{\sum }}{\left(x_{i}-\bar{x}\right)}^{4}}{{\left(1/N\sum_{i=1}^{N}{\left(x_{i}-\bar{x}\right)}^{2}\right)}^{2}}\text{.} \end{array}$$

In the frequency domain, 16 features are extracted manually. SNR is the ratio of signal power to noise power, where noise is measured by the RMS value. The signal-to-noise and distortion ratio is the ratio of total signal power to the total power of noise and distortion. THD is the ratio of total harmonic component power to fundamental component power.

MFCC is widely used as a signal feature in speech recognition tasks. Heart sound signals are also audio signals, so MFCC can be used as a feature to classify different categories of heart sound signals. To extract MFCC features, there are several steps, which are described as follows: firstly, a window technique such as hamming window should be performed to prevent spectral leakage. Secondly, a fast Fourier transform (FFT) is performed to get the spectrum of the signal. Thirdly, pass the spectrum through a set of triangle filters with the mel scale. The relation between the mel scale and frequency is defined in equation (4). Finally, we perform discrete cosine transform (DCT) of the mel spectrogram to get MFCCs. In this case, we preserve 13 coefficients to represent the signal.

(4) $$\begin{array}{c} Mel\left(f\right)=2595*\;\log\;\left(1+\frac{f}{700}\right)\text{.} \end{array}$$

#### 3.2.2. Feature Selection

Feature selection is quite important in machine learning because it can reduce the dimensions of the extracted features and have a relatively small computational load. Moreover, some features are redundant and make little contribution to the model. The performance of the model will probably improve after selecting the proper features. In this study, the chi-square test is used to test whether there is a significant difference between the expected frequencies and the observed frequencies of extracted features in different categories.

Chi-square test is one of the most widely used nonparametric test, which is used for data not satisfied with the parametric test such as normal distribution. The chi-square test will give the *p* value based on the degree of freedom and the chi-square value, where the degree of freedom equals the number of categories minus one.

**Figure 5:**
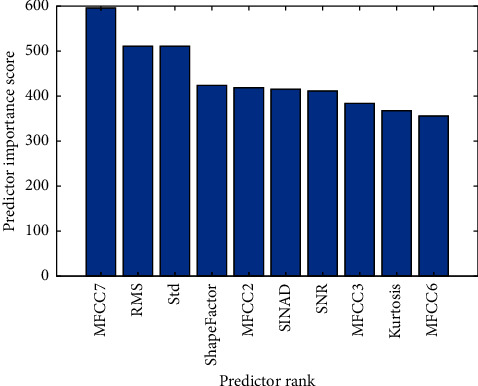
Predictor importance score of top ten important features.

**Figure 6:**
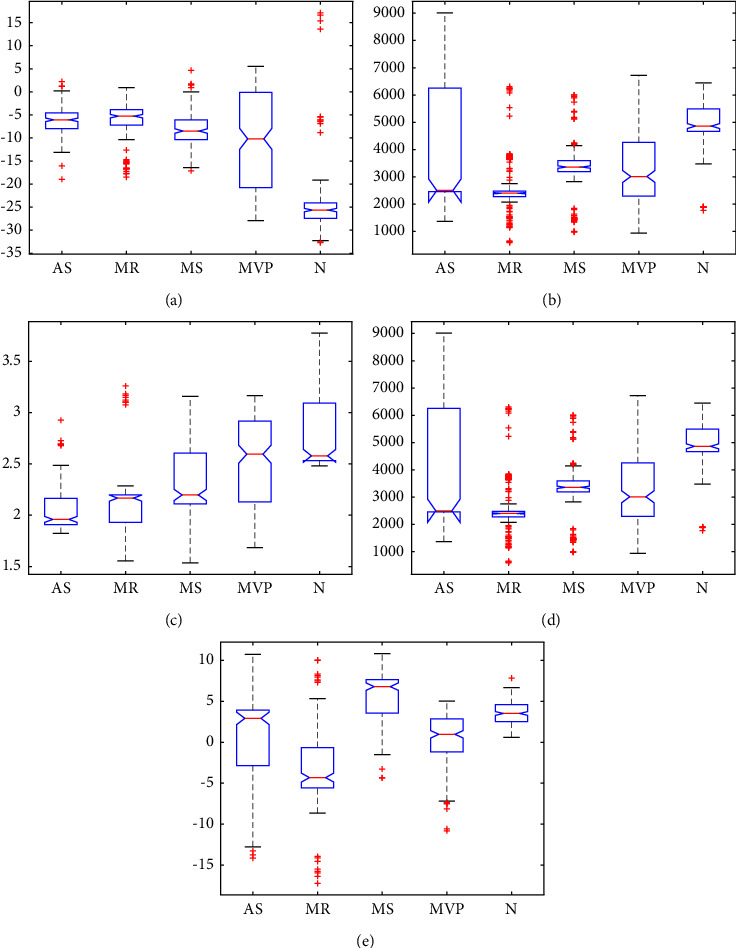
Boxplot of top five important features. (a) The 7^th^ MFCC. (b) RMS. (c) Std. (d) Shape factor. (e) 2^nd^ MFCC.

The predictor importance score is calculated by the *p* value shown in equation (5). The smaller the *p* value, the higher the importance score. The *p* value shows the significance between the features and categories. Features with higher importance scores have higher importance to the model. The predictor importance scores of the top ten important features are shown in Figure 5. The boxplots of the top five important features are shown in Figure 6. It can be found that the distribution of those features is quite different among different categories. Finally, fifteen features with the highest predictor importance score were selected.

(5) $$\begin{array}{c} Predictor\;importance\;score=-\ln\left(p\right)\text{.} \end{array}$$

#### 3.2.3. Classification with Weighted *K*-Nearest Neighbour

KNN is a nonparametric machine learning algorithm, which was developed by Evelyn Fix and Joseph Hodges. It is usually used for supervised learning. In KNN classification, the distances between the predicted sample and each sample in the training dataset are calculated and sorted. The predicted sample will be classified into the category by majority voting among its *k*-nearest neighbours.

WKNN is a modified version based on KNN. KNN classification has a shortage on the skewed dataset, which means examples in a more frequent class will be more common among the *k*-nearest neighbours. WKNN is designed to overcome this problem by giving a weight of 1/*d* to the distance, where *d* is the distance to the neighbours. Therefore, a longer distance will have a smaller weight, while a shorter distance will have a bigger weight.

**Figure 7:**
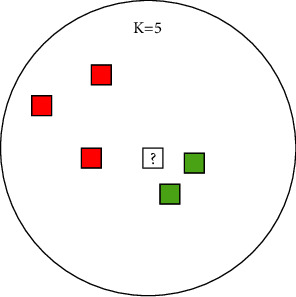
A misclassified example of KNN.

An example of misclassified by the KNN algorithm is shown in Figure 7. Intuitively, the predicted sample (white square) belongs to the green category. However, if we choose *k* equal to five, the predicted sample will be classified in the red category by voting among the five nearest neighbours, because the number of red samples is bigger than the green ones. After assigning the weight of distance, the distance from the green samples will have larger weights, and the predicted sample will be classified in the right category.

### 3.3. Model Assessment

Four model assessment indicators are used to evaluate the performance of our model, which are accuracy, specificity, sensitivity, and *F*1 score [49]. Accuracy can reflect the overall accuracy rate of the model. Specificity can reflect the level of misdiagnosis where the higher specificity corresponds to the lower misdiagnosis rate. Sensitivity can reflect the level of detection of the patients; the higher the sensitivity, the more patients the model can detect. The *F*1 score is a harmonic mean of specificity and sensitivity, which combines the information of both specificity and sensitivity. A higher *F*1 score corresponds to a higher value of both specificity and sensitivity.

The equations of those four indicators are as follows: a true positive means the person with the disease gets a positive result. A true negative means the person without disease gets a negative result. False-positive means the person without disease but gets a positive result. False negative means the person with the disease but gets a negative result.

(6) $$\begin{array}{c} Accuracy=\frac{True\;Positive+True\;Negative}{True\;Positive+True\;Negative+False\;Positive+False\;Negative}\text{,}\\ Specificity=\frac{True\;Negative}{True\;Negative+False\;Positive}\text{,}\\ Sensitivity=\frac{True\;Positive}{True\;Positive+False\;Negative}\text{,}\\ F1\;score=\frac{2*Specificity*Sensitivity}{Specificity+Sensitivity}\text{.} \end{array}$$

### 3.4. Heart Valve Disease Diagnosis System

#### 3.4.1. Design of Heart Sound Acquisition Module

**Figure 8:**
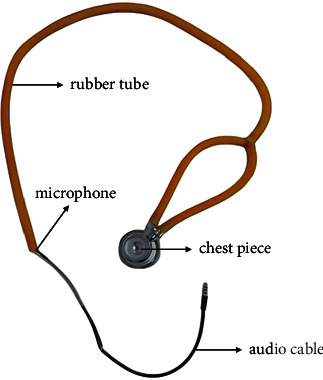
Structure of heart sound acquisition module.

A heart sound acquisition module was designed and made to acquire heart sounds from clinical, which contains a chest piece, rubber tubes, and a 3.5 mm microphone with audio cables connected to computers. The structure of the designed module is shown in Figure 8. The chest piece is a kind of resonator, which can nonlinearly amplify the sound generated by the heart valves and transmitted by the rubber tubes. The sound signal will be acquired through the microphone and imported into the diagnosis system to classify whether it is a normal heart sound or a kind of valvular heart disease.

#### 3.4.2. Software of the Diagnosis System

**Figure 9:**
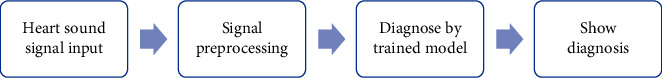
Heart valve disease diagnosis system.

After training and selecting the best model for classifying heart sounds, a heart valve disease diagnosing system is designed to meet the requirements of clinical application. The framework of the diagnosing system is shown in Figure 9. The software of the diagnosis system was designed based on MATLAB GUI (Graphical user interface) and converted to an EXE file, which can be installed and executed on different terminals of Windows with or without MATLAB software, which is robust and easy to use.

**Figure 10:**
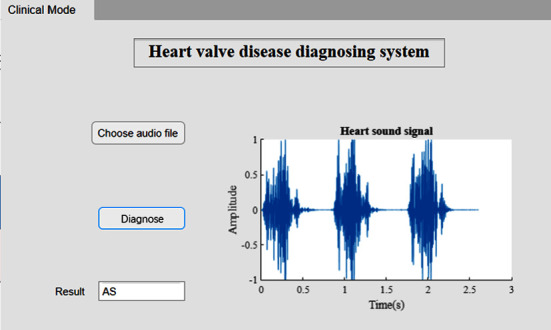
Software of heart valve disease diagnosing system (an example of aortic stenosis).

Once the audio file of a heart sound signal is chosen, the waveform of the signal will be displayed on the right side of the app, which makes the heart sound more intuitive. Then click the “Diagnose” button, and the program will preprocess the input signal, including downsampling or resampling the signal to 8000 Hz, which is identical to the training set, and convert the signal to its scalogram. Then the scalogram will be classified by the trained GoogLeNet model. Finally, the result of the diagnosis will be shown on the app.  Figure 10 shows the interface of the diagnosing system. Classification of aortic stenosis heart sounds was shown as an example.

## 4. Results

**Figure 11:**
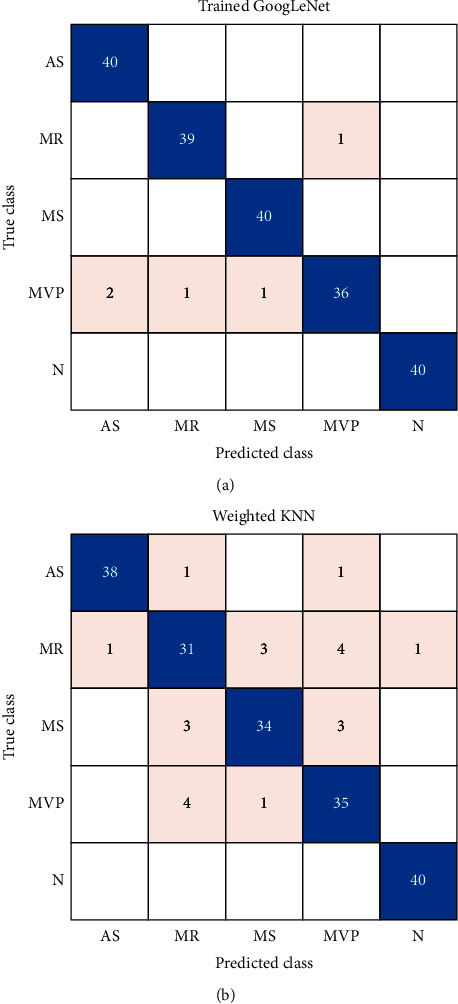
Confusion matrix of two models. (a) GoogLeNet. (b) WKNN.

Figure 11 shows the confusion matrix for the validation set, which contains 200 heart sound signals from two models trained by GoogLeNet and WKNN. The blue grids are the ones correctly classifying cases, while the pink grids are misclassifying cases. Most heart sounds are correctly classified.

Table 3 shows the indicators of model assessment for two trained models. Accuracy, sensitivity, specificity, and *F*1 score are calculated to assess the diagnostic performance of different kinds of heart valve diseases separately.

The model trained by GoogLeNet can perfectly identify healthy people and patients with valvular disease. For diagnosing four kinds of heart valve disease, the average accuracy, sensitivity, specificity, and *F*1 score are 98.75%, 96.88%, 99.22%, and 97.99%, respectively.

The model trained by WKNN also has high accuracy in diagnosing heart valve disease but is a little lower than the trained GoogLeNet model. The average accuracy, sensitivity, specificity, and *F*1 score for classifying four valvular diseases are 94.63%, 86.25%, 96.72%, and 91.11%, respectively.

## 5. Evaluation of Heart Valve Disease Diagnosing System

**Figure 12:**
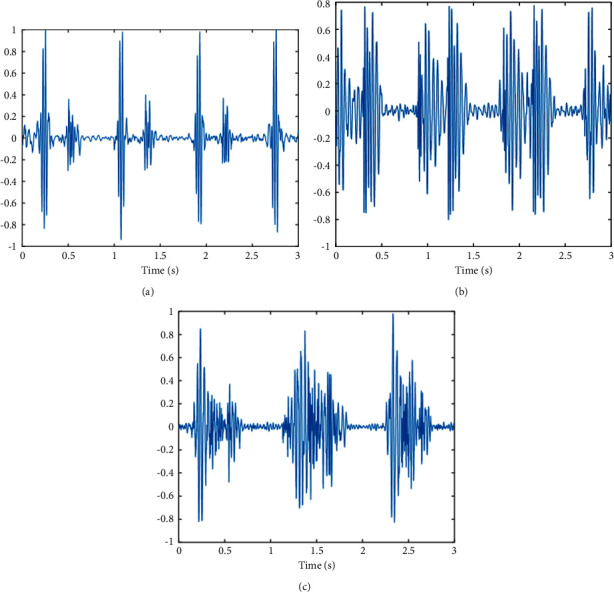
(a) Normal heart sound. (b) MR heart sound. (c) AS heart sound.

Eighteen pieces of heart sounds are recorded and downsampled to 8000 Hz from healthy people and valvular disease patients, which contain six pieces of normal heart sounds and twelve pieces of heart sounds from valvular diseases. Normal heart sounds were recorded from the mitral valve area of six healthy participants. Twelve pieces of valvular disease heart sounds are recorded from four participants, two each in MR and AS. Heart sounds of each kind of valvular disease are collected from three auscultation areas of each participant, which are the mitral valve area, the Erb area, and the aortic valve area. Three examples of each category of heart sounds are shown in Figure 12.

Import the audio files into the diagnosis system. The time-frequency scalograms are generated based on CWT. Then, a GoogLeNet model, which has been trained by scalograms of heart sounds, is used to classify imported heart sounds. The trained model correctly classified five normal heart sounds and eleven heart sounds associated with valvular diseases. Thus, an overall accuracy value of 88.89% is obtained through the diagnosing system. The classification accuracy of normal and valvular diseases is 83.33% and 91.67%, respectively.

## 6. Discussion and Conclusion

In this study, both models trained by the convolutional neural network GoogLeNet and the classic machine learning algorithm WKNN had high accuracy in separating healthy people from heart valve disease patients. In terms of detecting valvular heart disease, it is shown that the model trained by GoogLeNet has better performance than the model trained by WKNN by comparing the four indicators, which are accuracy, sensitivity, specificity, and *F*1 score, especially in diagnosing mitral regurgitation. In addition to the trained model, we proposed a whole heart valve disease diagnosis system. Heart sounds can be acquired by the heart sound acquisition module and diagnosed by uploading the recorded heart sound. Moreover, we collected three kinds of heart sounds, which are normal, MR, and AS, from valvular disease patients to verify our diagnosing system, and the experiment shows that it reached a high accuracy.

The proposed diagnosis system can collect heart sounds and diagnose four categories of valvular heart disease with high accuracy, which can assist doctors in diagnosing heart valve diseases and may greatly improve the accuracy of diagnosis in remote areas, which lack skilled cardiologists. In the future, we can collect more categories of heart sounds to train the model to diagnose more types of cardiovascular diseases, which may have a significant impact on reducing the uneven distribution of medical resources.

## Figures and Tables

**Table 1:** Related works on heart sound classification.

Year	Related work	Dataset	Classes	Feature extraction	Algorithm	Accuracy (%)
2021	Singh et al. [17]	PhysioNet 2016	2	Wavelet decomposition, homomorphic filtering, Hilbert transform, and power spectral density without segmentation	AlexNet	90.00
2020	Krishnan et al. [15]	PhysioNet 2016	2	Feedforward neural network	Feedforward neural network	85.65
2020	Hu et al. [18]	PhysioNet 2016	2	1D-CNN	ANN	94.60
2021	Zeng et al. [19]	PhysioNet 2016	2	Tunable Q-factor wavelet transform, variational mode decomposition, phase space reconstruction	Radial basis neural network	97.89
2021	Tuncer et al. [20]	Yaseen dataset	5	Petersen graph pattern	KNN, DT, LD, BT, SVM	KNN: 100.00 DT: 95.10 LD: 98.30 BT: 98.60 SVM: 99.90
2018	Yaseen et al. [21]	Yaseen dataset	5	MFCC and DWT	SVM, KNN, and DNN	SVM: 97.90 KNN: 97.40 DNN: 92.10
2020	Chen et al. [22]	PhysioNet 2016	2	Modified frequency slice wavelet transform	CNN	94.00
2016	Abdollahpur et al. [23]	PhysioNet 2016	2	Shannon entropy	5 groups of features from the time-domain, time-frequency domain, and perceptual features such as Shannon entropy, MFCC, etc	92.48
2020	Ghosh et al. [24]	Yaseen dataset	4	Chirplet transform, local energy, and local entropy	Sparse representation classifier	98.54
2020	Ghosh et al. [25]	Yaseen dataset	5	Spline kernel-based chirplet transform, *L*1-norm, sample entropy, and permutation entropy	Deep layer kernel sparse representation network	95.67
2019	Ghosh et al. [26]	Yaseen dataset	4	Statistical features extracted from time-frequency magnitude and phase matrix of segmented PCG signals	Random forest	93.91
2022	Karhade et al. [27]	Yaseen dataset and PhysioNet 2016	4	Time-frequency images obtained using both time domain polynomial chirplet transform (TDPCT) and frequency-domain polynomial chirplet transform (FDPCT)	Deep convolutional neural network	TF images obtained from TDPCT: 99.00 TF images obtained from FDPCT: 99.48
2019	Singh and Majumder [28]	PhysioNet 2016	2	Wavelet decomposition, Hilbert transform, homomorphic filtering, and power spectral density	KNN	90
2020	Yang et al. [29]	PASCAL and PhysioNet 2016	2	Time domain features based on envelope extracted signal processed by EMD	SVM	96.67
2021	Tseng et al. [30]	PhysioNet 2016	2	—	Booster LKNet	92.48
2021	Alkhodari and Fraiwan [31]	Yaseen dataset	5	—	CNN-BiLSTM	99.32
2021	Sankararaman [32]	48 mitral incompetence and healthy heart sound signals	2	Wavelet scalogram generated by CWT	KNN	100
2018	Latif et al. [33]	PhysioNet 2016	2	MFCC from 25 ms of the window with a step size of 10 ms	RNN with BiLSTM units	98.61
2017	Zhang and Wei [34]	PhysioNet 2016	2	20 waveform features extracted from segmented signals and 15 power spectral density features extracted from several frequency ranges	Least squares support vector machine (LS-SVM)	86.85
2022	Morshed et al. [35]	PhysioNet 2016 and Yaseen dataset	2/5	Magnitude, frequency, and phase of each Burg's spectrum along with statistical features	Ensembled bagged trees	Yaseen dataset: 99.28 PhysioNet 2016: 93.46
2022	Tariq et al. [36]	PASCAL dataset	6	Spectrogram and chromagram	FDC-FS network	97.00
2022	Sun et al. [37]	665 AR, 381 AS, 315 ASD, 769 MR, 439 MS, 1056 NM and 327 VSD sounds	7	Frequency features extracted from envelopes of segmented signal processed by short-time modified Hilbert transform	Squared mahalanobis distance classification	99.43, 98.93, 99.13, 99.85, 98.62, 99.67 and 99.91 in the detection of MR, MS, ASD, NM, AS, AR and VSD, respectively
2022	See et al. [38]	PhysioNet 2016	2	Shannon entropy and spectral entropy from three frequency bands	SVM	82.50
2022	Zhou et al. [39]	PhysioNet 2016	2	—	Dense feature selection convolution network	86.70
2021	Shuvo et al. [40]	Yaseen dataset	5	Convolutional layers and max-pooling layers to extract time, frequency, and pattern features	CardioXNet (A CRNN network, including representation learning and sequence residual learning)	88.09
2021	Gelpud et al. [41]	PhysioNet 2016	2	CWT scalogram of segmented sounds based on teager energy operator and autocorrelation	ResNet152 and VGG16	ResNet152: 91.19 VGG16: 90.75
2022	Arshad et al. [42]	PhysioNet 2016	2	Mean value of each window of short-time power spectral density	Decision tree	84.94
2021	Duggento et al. [43]	PhysioNet 2016	2	MFCC	Ad hoc multibranch, multiinstance artificial neural network	97.00
2022	Tian et al. [44]	PhysioNet 2016	2	Extract gramian angular fields image features by ResNet2 and extract sequence features by MobileNet-LSTM	Combination of ResNet2 and MobileNet-LSTM	97.99

1D-CNN: one dimension convolution neural network; ANN: artificial neural network; KNN: *K*-nearest neighbours; DT: decision tree; LD: linear discriminant; BT: bagged trees; SVM: support vector machine; MFCC: mel-frequency cepstral coefficients; DWT: discrete wavelet transform; TDPCT: time domain polynomial chirplet transform; FDPCT: frequency-domain polynomial chirplet transform; EMD: empirical mode decomposition; BiLSTM: bidirectional long short-term memory; CWT: continuous wavelet transform; RNN: recurrent neural network; LS-SVM: least squares support vector machine; FDC-FS: fusion-based disease classification-fusion; ASD: atrial septal defect; VSD: ventricular septal defect; NM: normal.

**Table 2:** List of extracted features.

No.	Features	Domain (time or frequency)
1	Root mean square (RMS)	Time domain
2	Shape factor	
3	Skewness	
4	Kurtosis	
5	Peak value	
6	Impulse factor	
7	Crest factor	
8	Clearance factor	
9	Mean	
10	Standard deviation (std)	

11	Signal-to-noise ratio (SNR)	Frequency domain
12	Signal-to-noise and distortion ratio	
13	Total harmonic distortion (THD)	
14–26	Mel frequency cepstral coefficients (MFCC1-MFCC13)	

**Table 3:** (a). Model assessment indicators of the trained GoogLeNet model. (b) Model assessment indicators of the trained WKNN model.

Indicators	AS (%)	MR (%)	MS (%)	MVP (%)	*N* (%)
*(a)*					
Accuracy	99.00	99.00	99.50	97.50	100.00
Sensitivity	100.00	97.50	100.00	90.00	100.00
Specificity	98.75	99.38	99.38	99.38	100.00
*F*1 score	99.37	98.43	99.69	94.46	100.00

*(b)*					
Accuracy	98.50	91.50	95.00	93.50	99.50
Sensitivity	95.00	77.50	85.00	87.50	100.00
Specificity	99.38	95.00	97.50	95.00	99.38
*F*1 score	97.14	85.36	90.82	91.10	99.69

## Data Availability

The data used to support the findings of this study are available from the corresponding author upon request.
